# Baseline Plasma Fatty Acids Profile and Incident Cardiovascular Events in the SU.FOL.OM3 Trial: The Evidence Revisited

**DOI:** 10.1371/journal.pone.0092548

**Published:** 2014-04-07

**Authors:** Léopold K. Fezeu, François Laporte, Emmanuelle Kesse-Guyot, Valentina A. Andreeva, Jacques Blacher, Serge Hercberg, Pilar Galan

**Affiliations:** 1 Université Paris 13, Sorbonne Paris Cité - Equipe de Recherche en Epidémiologie Nutritionnelle (EREN), Centre d'Epidémiologie et Biostatistiques (EPIBIOS), Inserm, Inra, Cnam, Université Paris 5, Université Paris 7, Bobigny, France; 2 Département de Biochimie–Pharmacologie–Toxicologie, Centre Hospitalier Universitaire, Grenoble, France; 3 Université Paris-Descartes, Faculté de Médecine, AP-HP; Hôtel-Dieu, Centre de Diagnostic et Thérapeutique, Paris, France; 4 Département de Santé publique, Hôpital Avicenne, Bobigny, France; Scuola Superiore Sant'Anna, Italy

## Abstract

**Objective:**

We aimed to investigate the association between baseline plasma fatty acids profile and the risk of future major cardiovascular events in patients with a history of ischaemic heart disease or ischemic stroke.

**Methods:**

Baseline plasma fatty acids as well as established cardiovascular risk factors were measured in 2,263 patients enrolled in the SUpplementation with FOLate, vitamins B-6 and B-12 and/or OMega-3 fatty acids randomized controlled trial. Incident major cardiovascular, cardiac and cerebrovascular events were ascertained during the 4.7 years of follow up. Hazard ratios were obtained from Cox proportional hazards models after adjustment for cardiovascular risk factors.

**Results:**

During the follow-up, 154, 379 and 84 patients had major cardiovascular, cardiac and cerebrovascular events respectively. Upon adjustment for gender, initial event, baseline age and BMI, the risk of developing a major cardiovascular event decreased significantly in successive quartiles of arachidonic acid (P_trend_<0.002), total omega 3 polyunsaturated fatty acids (P_trend_<0.03), docosapentaenoic acid (P_trend_<0.019), docosahexaenoic acid (P_trend_<0.004), eicosapentaenoic acid + docosahexaenoic acid (P_trend_<0.03) and eicosapentaenoic acid + docosapentaenoic acid + docosahexaenoic acid (P_trend_<0.02). This inverse association was borderline significant with increased quartiles of stearidonic acid (P_trend_<0.06). In the full model, only stearidonic acid remained inversely associated with the risk of developing a major cardiovascular event (P_trend_<0.035), a cardiac event (P_trend_<0.016) or a cerebrovascular event (P_trend_<0.014), while arachidonic acid was inversely associated with the risk a cerebrovascular event (P_trend_<0.033).

**Conclusion:**

The inverse association of long chain omega 3 polyunsaturated fatty acids with recurrence of Cardiovascular diseases was mainly driven by well-known cardiovascular risk factors.

**Trial Registration:**

Controlled-Trials.com ISRCTN41926726

## Introduction

The incidence of cardiovascular diseases (CVD) is suggested to be modulated by dietary fatty acid composition, ie saturated fatty acids (SFA) increasing and polyunsaturated fatty acids (PUFA) decreasing serum cholesterol levels and risk of CVD [1]. Some fatty acids influence the incidence of CVD through triglyceride accumulation, inflammation, vasodilation, or platelet aggregation [2]. SFA and monounsaturated fatty acids (MUFA) are the main components of triglycerides, although mechanisms linking the relationships between CVD and individual fatty acids remain uncertain.

Based on these hypotheses, several epidemiological studies have examined associations between several fatty acids and stroke, arrhythmia and coronary disease risk factors. Some prospective epidemiologic studies have reported a significant association between fish intake or n–3 fatty acids derived from fish oil and a lower risk of CVD [1], [3]–[6]. However, randomized controlled trials and meta-analyses have yielded inconsistent results [1], [7]–[12]. A number of hypotheses could explain these discrepancies. First, the changes in tissue lipid composition as well as the beneficial anti-inflammatory and anti-atherogenic properties of a diet high in n–3 fatty acids require a long time to express. Thus, studies and meta-analyses conducted for less than one year may be unable to detect a clinical benefit [12]. Second, supplements given as part of dietary interventions have a strictly controlled dose and content unlike others; therefore, while compliance with a “self-administered” dietary supplement may be greater than that in dietary interventions, it is not clear whether both supplementations have similar effects. Many systematic reviews have included both dietary interventions and dietary supplements; this is likely to lead to significant heterogeneity [11]. Equally important is the fact that different formulations with different dosages have been used. As α-linolenic acid is a plant-based n–3 polyunsaturated fatty acid (PUFA) that is poorly converted (less than 5%) to eicosapentaenoic acid (EPA) and docosahexaenoic acid (DHA), it is therefore unlikely that it would have the cardioprotective effects of fish oil [13], [14].

Although several studies have examined the relationship between PUFA intake and the incidence of CVD, no clear consensus yet exists regarding the effects of various fatty acid compositions, including that of PUFA on the development of CVD [7], [8], [15]. This may be at least partly due to the fact that dietary intake is difficult to quantify precisely due to self-reporting bias, difficulties in evaluating consumed portion sizes, lack of information about cooking methods, and lack of precision as regards food composition tables, particularly for processed food [16], [17]. Usual methods of dietary assessment such as food frequency questionnaires, diet history or repeated 24-h recall/records are used in large epidemiological studies to assess fatty acids intake, yet these are subject to the above limitations, as well as to errors in recall [16], [17].

We recently completed the SU.FOL.OM3 study, a double blind, randomized, placebo controlled secondary prevention trial which aimed to investigate whether dietary supplementation with B vitamins or n–3 fatty acids, or both, could prevent subsequent major cardiovascular events in patients with a history of CVD [18]. Supplementation with n-3 fatty acids did not have any significant effects on major vascular events. In the present ancillary analysis of the SU.FOL.OM3 trial, we explored the association between baseline plasma fatty acids (PFA) and the risk of secondary cardiovascular events in this population with recent medical history of CVD.

## Methods

The supporting CONSORT checklist is available as supporting information; see Checklist S1.

### Ethics statement

The protocol was approved by the ethic commitee “Comité Consultatif pour la Protection des Personnes se prêtant à la Recherche Biomédicale” (CCPPRB n° 1933) of Paris-Cochin Hospital, and the data protection board “Commission Nationale de l'Informatique et des Libertés” (CNIL n° 901230). Participants provided their written informed consent to participate in this study. The current analyses included 2,263 subjects for whom baseline PFA were available.

### Study population

Details concerning study methods and participant characteristics were reported elsewhere [18]. In brief, 2,501 participants 45–80 years (1,987 men and 514 women) with a prior history of an acute coronary or cerebral ischemic event occurring within 1 to 12 months were recruited via a network of 417 cardiologists, neurologists or other physicians throughout France. They were randomly assigned using a two-by-two factorial design, to receive either 1) B-vitamins alone, 2) n-3 PUFA alone 3) both active treatments or 4) both placebo treatments.

### CVD ascertainment

Every six months, questionnaires were mailed to all participants seeking information about any serious disease outcomes, compliance with the assigned regimen, presence of cardiovascular risk factors and adverse effects of the supplementation. A non-mandatory visit was scheduled annually and participants who were unable or unwilling to attend or who did not return the questionnaire were interviewed by telephone by study physicians. Family physicians also contacted the trial participants every six months in order to seek information on any health events that may have occurred. Two independent committees of cardiologists or neurologists who were blinded to the treatment allocation adjudicated all events. Participants were censored either on the date of their first major event, the date of death, or, for those without an event, at the end of follow-up on July 1, 2009. Three composite endpoints were used to evaluate the association between baseline PFA and cardiovascular events: 1) Major cardiovascular events, which included non-fatal myocardial infarction, non-fatal ischemic stroke or death from cardiovascular disease. 2) Cardiac events, which included acute coronary syndrome without myocardial infarction; resuscitation from sudden death; coronary artery bypass surgery; coronary angioplasty; cardiac failure; ventricular arrhythmia; supraventricular arrhythmia; cardiac surgery of any kind and carotid surgery or caro0tid artery angioplasty. 3) Cerebrovascular events, which included: ischemic or haemorrhagic stroke and transient ischemic attack.

### Assessment of PFA profile and data collected at baseline

Data on gender, age, smoking status, existing hypertension, diabetes or dyslipidaemia, n-3 supplementation and medication use were collected at baseline via a questionnaire. Dietary intake (especially fish consumption) and alcohol consumption were assessed using a Food Frequency Questionnaire. Body mass index (BMI in kg/m^2^) was calculated from the standardized measurements of height and weight. Blood pressure was recorded by trained staff for each patient in the sitting position, after a 5-min rest.

Venous blood samples were drawn after at least a 5-hour fast. Measured biological parameters included cholesterol (total, LDL, and HDL) and triglycerides (measured by enzymatic method with the use of C8000 System Abbott, Rungis, France), fasting blood glucose concentrations (enzymatic method) and total homocysteine (measured by a competitive immunoassay with direct chemiluminescence detection). Biological assessments were obtained by standard methods at a centralized laboratory.

Total (free and esterified) fatty acids composition of plasma lipids was determined by gas chromatography of the corresponding methyl esters. Cleavage of the ester bonds of triglycerides and cholesterol esters was performed by saponification. Specifically, lipids were extracted from 150 µL aliquots of plasma with 8 mL hexane: isopropanol (3∶2, vol∶vol) after the addition of heptadecanoic acid as an internal standard. The fatty acids were saponified with 1.0 mL NaOH in dry methanol at 100°C for 10 min and methylated with boron trifluoride 14% in methanol. The methylated fatty acids were extracted with n-heptane and quantified by gas chromatography with a flame ionisation detector (6850 Agilent) using a capillary column (Quadrex 30 m length, 0.25 mm id, film thickness 0.25 µm, Alltech). The temperature gradient was programmed at 50°C/min from 50°C to 140°C, 1.4°C/min from 140°C to 165°C and 10°C/min from 165°C to 245°C/min. Hydrogen was the carrier gas. The identification of the individual methyl ester components was made by comparison with a mixture of commercial standards. The results were expressed as percentages of the total area of all fatty acid peaks.

### Statistical analyses

Data are expressed as mean (±SD) or median (25^th^–75^th^ percentiles) and percentages (95% CI) as appropriate. Comparison of baseline characteristics between participants with and without a recurrent cardiovascular event relied on Student t tests, Wilcoxon Rank tests or Chi square tests as appropriate. Cox proportional hazards models were used to estimate the hazard ratios (HRs) and 95% 95% CI for major cardiovascular, cardiac and cerebrovascular events by quartiles of each PFA as well as total saturated fatty acids (SFA), trans fatty acids, monounsaturated fatty acids (MUFA), PUFA, n-6 PUFA, n-3 PUFA, EPA + DPA, EPA + DPA + DHA, and the n-3 PUFA to n-6 PUFA ratio. Associations with baseline plasma fatty acids were adjusted for two sets of potential confounding factors in model 1 (initial event, gender, baseline age and BMI) and model 2 (model 1 + treatment allocation group (both placebos, B vitamin and placebo of omega 3, omega 3 and placebo of B vitamins, omega 3 and B vitamins), tobacco smoking, alcohol consumption, diabetes status, systolic blood pressure, plasma cholesterol (total, HDL, LDL), n-3 supplementation, homocystinemia and total fatty acids intake). Statistical analyses were performed using STATA 10. Statistical tests were two-sided and p-values<0.05 were considered to be statistically significant.

## Results

During the follow-up, among the 2,263 participants with available PFA, 154 developed a major cardiovascular event (median time to event, 2.3 years, 25^th^–75^th^ percentiles: 1.3–3.3), 379 a cardiac event (1.3 years, 0.3–2.7) and 85 a cerebrovascular event (2.2 years, 1.2–3.2). The median follow-up of participants who did not develop a cardiovascular event until the end of the study was 4.8 (3.9–5.0) years. The major cardiovascular events included 57 stroke cases (among which 52 were non-fatal), 19 deaths from cardiac origin, 56 non-fatal myocardial infarctions and 14 sudden cardiac deaths. Cardiac events included 122 angioplasties, 51 myocardial infarctions, 45 cardiac arrhythmias, 40 coronary revascularisations and 40 cardiac interventions. Cerebrovascular events included 52 non-fatal ischemic stroke and 20 transient ischemic attacks.

### Baseline characteristics

The mean baseline age of the participants was 61.6 (range: 41.9–81.2) years, and 80.6% of them were men. Regardless of the outcome considered, patients who developed a cardiovascular event more often had diabetes and were current smokers at baseline (except for cardiac events, Table 1). Participants with major cardiovascular events or with cardiac events had higher plasma triglycerides, fasting plasma glucose and homocysteinaemia.

**Table 1:** Baseline sociodemographic, clinical and biologic characteristics of SU. FOL.OM3 participants without and with major events, cardiac and cerebrovascular events at the end of the follow-up.

Characteristics	No events	Major events	P*	Cardiac events	P†	Cerebrovascular events	P‡
N	1717	154		379		85	
EPA + DPA supplementation, n (%)	1717 (50.9)	78 (50.6)	0.95	185 (48.8)	0.46	40 (47.1)	0.49
Age, years	61.4±8.9	63.6±9.5	**0.006**	62.3±9.3	0.12	66.0±8.5	**<0.0001**
Men, n (%)	1375 (80.1%)	124 (80.5%)	0.67	315 (83.1%)	0.13	69 (81.2%)	0.43
BMI, kg/m^2^	27.5±4.0	27.8±4.4	0.27	27.7±4.0	0.48	28.0±4.1	0.19
SBP, mm Hg	133.1±21.3	136.8±23.4	0.24	134.1±22.0	0.78	141.5±23.9	**0.03**
DBP, mm Hg	83.4±12.2	83.4±12.5	0.87	82.5±12.5	0.16	85.5±12.2	0.20
MAP, mm Hg	99.9±14.5	101.2±15.3	0.64	99.7±14.8	0.51	104.2±15.3	0.08
Total energy intake, kcal/day§	2445±991	2343±856	0.50	2470±941	0.52	2299±591	0.61
Total fibre intake, g/day§	24.6±10.7	23.2±9.8	0.13	25.2±10.6	0.42	24.0±9.3	0.52
Total fish intake, g/day§	62.3±63.9	51.6±39.6	0.07	63.7±70.8	0.67	51.1±44.4	0.20
Fat fish intake, g/day§	19.7±26.9	17.3±21.4	0.40	18.9±22.0	0.65	17.0±20.3	0.55
Total cholesterol, mmol/L	4.60±1.05	4.78±1.03	**0.04**	4.68±1.09	0.24	4.88±1.10	**0.03**
HDL-cholesterol, mmol/L	1.20±0.30	1.21±0.32	0.57	1.19±0.32	0.22	1.24±0.34	0.85
LDL-cholesterol, mmol/L	2.76±0.84	2.88±0.86	0.11	2.84±0.90	0.19	2.99±0.91	**0.03**
Triglycerides, mmol/L	1.40±0.77	1.60±1.00	**0.004**	1.50±0.96	**0.04**	1.46±0.88	0.36
Glycaemia, mmol/L	5.81±1.56	6.19±2.09	**0.009**	6.12±2.09	**0.001**	5.94±1.80	0.51
Smoking status, n (%)			**0.007**		0.23		**0.002**
Current smoker	181 (10.7)	25 (16.3)		36 (9.5)		14 (16.7)	
Former smoker	1040 (61.5)	92 (60.1)		249 (65.7)		52 (61.9)	
Never smoked	471 (27.8)	36 (23.5)		94 (24.8)		18 (21.4)	
Alcohol intake, g/day			**0.002**		0.16		**0.007**
>10	833 (48.5)	71 (46.1)		181 (47.8)		40 (47.1)	
0.5–10	468 (27.3)	27 (17.5)		88 (23.2)		13 (15.3)	
<0.5	156 (9.1)	19 (12.3)		41 (10.8)		11 (12.9)	
Missing information	260 (15.1)	37 (24.0)		69 (18.2)		21 (24.7)	
Inclusion criteria, n (%)			**0.009**		**<0.0001**		**<0.0001**
Stroke	444 (25.9)	59 (38.3)		55 (14.5)		52 (61.2)	
Myocardial infarction	773 (45.0)	63 (40.9)		198 (52.2)		18 (21.2)	
Unstable angina	500 (29.1)	32 (20.8)		126 (33.2)		15 (17.6)	
Diabetes			**0.004**		**0.002**		**0.03**
Yes	278 (16.2%)	40 (26%)		87 (23.0%)		22 (25.9%)	
No	1439 (83.8%)	114 (74%)		292 (77.0%)		63 (74.1%)	

Data are frequencies (percentages) or mean ± SD.

P values are for the comparison of ^*^major, †cardiac and ‡cerebrovascular events with participants without events at the end of the study.

§ Food frequency questionnaires were filled only by 85% of the study participants, and these values are obtained from subjects for which the information was available.

### Baseline PFA and cardiovascular events

In bivariate analyses, stearidonic acid was lower in patients who developed a cardiovascular event, regardless the event considered. Total fatty acids were higher and arachidonic acid, total n–3 PUFA, DPA, DHA and the sum of EPA + DPA + DHA were lower in patients who developed a major cardiovascular event (Table 2). Arachidonic acid was lower in patients who developed a cerebrovascular event.

**Table 2:** Baseline plasma lipid profile of SU.FOL.OM3 participants without and with major events, cardiac and cerebrovascular events at the end of the follow-up.

Plasma lipid profile	No events	Major events	P*	Cardiac events	P†	Cerebrovascular events	P‡
N	1717	154		379		85	
Total fatty acids, µmol/L	10568±2677	11302±2920	0.003	10756.3	0.24	11056.1	0.19
Total saturated FA	29.71±2.89	29.99±3.26	0.52	29.74±2.97	0.99	29.73±3.19	0.77
Palmitic acid (C 16:0)	22.28±2.28	22.55±2.47	0.31	22.33±2.37	0.85	22.38±2.34	0.90
Trans fatty acids	0.14±0.10	0.14±0.10	0.98	0.14±0.11	0.79	0.14±0.10	0.91
Total MUFA	25.83±3.73	26.51±3.71	0.10	25.89±3.79	0.90	26.21±3.67	0.97
Oleic acid (C 18:1 n – 9)	21.30±3.03	21.75±3.16	0.24	21.38±3.18	0.72	21.39±3.11	0.51
Total PUFA	44.13±5.66	43.18±5.64	0.16	44.05±5.65	0.96	43.73±5.46	0.90
Total n-6 PUFA	38.71±5.31	38.07±5.31	0.44	38.75±5.47	0.75	38.59±5.01	0.59
Linoleic acid (C 18:2 n – 6)	27.28±4.69	27.07±4.57	0.95	27.52±4.74	0.19	27.70±4.65	0.16
Dihomo-Υ-linolenic acid (C20:3 n – 6)	1.59±0.34	1.62±0.40	0.30	1.58±0.36	0.64	1.66±0.44	0.058
Arachidonic acid (C 20:4 n – 6)	8.79±1.96	8.34±2.06	0.04	8.60±2.15	0.06	8.22±1.99	0.04
Total n-3 PUFA	5.42±1.84	5.11±1.83	0.04	5.30±1.89	0.29	5.14±1.79	0.24
α-linolenic acid (C 18:3 n – 3)	0.61±0.27	0.59±0.24	0.44	0.62±0.27	0.17	0.57±0.20	0.41
Stearidonic acid acid (C 18:4 n – 3)	0.04±0.04	0.03±0.03	0.006	0.03±0.04	0.007	0.02±0.03	0.005
EPA (C 20:5 n – 3)	1.42±0.87	1.33±0.85	0.16	1.38±0.90	0.37	1.33±0.88	0.42
DPA (C 22:5 n – 3)	0.60±0.15	0.57±0.15	0.03	0.59±0.17	0.38	0.58±0.14	0.10
DHA (C 22:6 n – 3)	2.75±0.92	2.58±0.93	0.04	2.68±0.91	0.15	2.63±0.88	0.33
EPA + DHA	4.17±1.68	3.91±1.67	0.06	4.06±1.70	0.21	3.96±1.66	0.34
EPA + DPA + DHA	4.77±1.76	4.48±1.77	0.05	4.65±1.81	0.21	4.54±1.74	0.30
N – 3/n – 6 ratio	0.14±0.05	0.14±0.05	0.15	0.14±0.06	0.37	0.13±0.05	0.18

All values are expressed as percentage of total plasma fatty acids, except for total plasma fatty acids.

P values are for the comparison of ^*^major, †cardiac and ‡cerebrovascular events with participants without events at the end of the study, after adjusting for total energy intake.

In model 1, the risk of developing a major cardiovascular event increased significantly in successive quartiles of total fatty acids and decreased in successive quartiles of arachidonic acid, total n–3 PUFA, DPA, DHA, EPA + DHA and EPA + DPA + DHA (Table 3). This inverse association was borderline significant with increasing quartiles of stearidonic acid. The prevalence of major cardiovascular events was lower in the third and fourth quartiles of baseline plasma arachidonic acid (HR: 0.62, 0.40–0.96, p<0.03 and 0.51, 0.32–0.81, p<0.005 respectively) DPA (HR: 0.58, 0.37–0.91, p<0.019 and 0.64, 0.40–1.00, p<0.05 respectively) and in the fourth quartiles of baseline plasma DHA (p<0.001), EPA + DHA (p<0.046) and EPA + DPA + DHA (p<0.029) respectively, in comparison with the first quartile. Furthermore, the risk of developing a cardiac event decreased with increasing quartiles of stearidonic acid, EPA and DHA (Table 4). The risk of developing a cerebrovascular event decreased with increasing quartiles of arachidonic acid and stearidonic acid (Table 5).

**Table 3:** Multivariate hazard ratios (95% confidence intervals) showing the associations between baseline plasma lipid profile and incident major events in the SU.FOL.OM3 study.

Plasma lipid profile	HR Q1	HR Q2	HR Q3	HR Q4	P	P for trend
**Total fatty acids**						
Model 1	1.0	1.32 (0.80–2.17)	1.54 (0.95–2.50)	1.54 (1.08–2.81)	0.13	0.018
Model 2	1.0	1.18 (0.62–2.25)	1.37 (0.65–2.89)	1.06 (0.35–3.25)	0.68	0.15
**Total SFA**						
Model 1	1.0	0.56 (0.35–0.91)	0.81 (0.52–1.25)	0.97 (0.64–1.48)	0.08	0.75
Model 2	1.0	0.58 (0.33–1.01)	0.82 (0.49–1.37)	0.71 (0.39–1.30)	0.27	0.42
**Palmitic acid (C 16:0)**						
Model 1	1.0	0.58 (0.36–0.94)	0.88 (0.57–1.35)	0.94 (0.61–1.45)	0.14	0.80
Model 2	1.0	0.48 (0.27–0.86)	0.82 (0.49–1.37)	0.63 (0.34–1.17)	0.077	0.33
**Trans fatty acids**						
Model 1	1.0	0.99 (0.64–1.54)	0.95 (0.61–1.50)	0.92 (0.58–1.45)	0.98	0.69
Model 2	1.0	0.95 (0.57–1.58)	1.03 (0.61–1.75)	0.90 (0.52–1.56)	0.96	0.80
**Total MUFA**						
Model 1	1.0	1.74 (1.07–2.83)	1.52 (0.92–2.50)	1.88 (1.16–3.07)	0.066	0.029
Model 2	1.0	1.61 (0.91–2.84)	1.50 (0.83–2.71)	1.55 (0.79–3.04)	0.40	0.25
**Oleic acid (C 18:1 n – 9)**						
Model 1	1.0	1.06 (0.66–1.70)	1.28 (0.82–2.01)	1.29 (0.82–2.03)	0.59	0.19
Model 2	1.0	1.12 (0.65–1.91)	1.04 (0.60–1.82)	0.94 (0.49–1.81)	0.94	0.834
**Total PUFA**						
Model 1	1.0	0.79 (0.51–1.21)	0.83 (0.54–1.27)	0.64 (0.41–1.02)	0.31	0.089
Model 2	1.0	1.25 (0.71–2.21)	1.11 (0.59–2.09)	1.05 (0.53–2.08)	0.85	0.93
**Total n-6 PUFA**						
Model 1	1.0	0.91 (0.59–**1**.41)	0.87 (0.56–1.34)	0.71 (0.45–1.13)	0.53	0.15
Model 2	1.0	0.95 (0.54–1.67)	0.99 (0.54–1.80)	0.93 (0.49–1.77)	0.99	0.87
**Linoleic acid (C 18:2 n – 6)**						
Model 1	1.0	0.93 (0.58–1.48)	1.19 (0.77–1.85)	1.20 (0.77–1.88)	0.62	0.53
Model 2	1.0	0.96 (0.55–1.67)	0.99 (0.58–1.69)	1.34 (0.80–2.25)	0.55	0.28
**Dihomo-Υ-linolenic acid (C 20:3 n – 6)**						
Model 1	1.0	0.96 (0.61–1.51)	0.99 (0.64–1.59)	1.02 (0.65–1.61)	0.99	0.88
Model 2	1.0	1.15 (0.66–2.02)	1.32 (0.77–2.27)	1.43 (0.82–2.49)	0.61	0.18
**Arachidonic acid (C 20:4 n – 6)**						
Model 1	1.0	0.75 (0.49–1.12)	0.62 (0.40–0.96)	0.51 (0.32–0.81)	0.023	0.002
Model 2	1.0	0.91 (0.55–1.52)	0.84 (0.49–1.44)	0.60 (0.32–1.12)	0.424	0.133
**Total n-3 PUFA**						
Model 1	1.0	0.89 (0.59–1.35)	0.69 (0.44–1.07)	0.65 (0.41–1.03)	0.184	0.032
Model 2	1.0	1.46 (0.89–2.39)	0.85 (0.48–1.52)	0.83 (0.46–1.50)	0.103	0.253
**α-linolenic acid (C 18:3 n – 3)**						
Model 1	1.0	0.94 (0.61–1.45)	0.91 (0.59–1.43)	0.92 (0.59–1.44)	0.976	0.682
Model 2	1.0	0.96 (0.58–1.60)	0.88 (0.52–1.51)	0.89 (0.52–1.53)	0.989	0.612
**Stearidonic acid (C 18:4 n – 3)**						
Model 1	1.0	1.14 (0.76–1.71)	0.80 (0.53–1.23)	0.66 (0.40–1.09)	0.152	0.062
Model 2	1.0	0.93 (0.58–1.51)	0.72 (0.44–1.18)	0.56 (0.30–1.01)	0.206	0.035
**EPA (C 20:5 n – 3)**						
Model 1	1.0	0.95 (0.62–1.45)	0.83 (0.54–1.29)	0.78 (0.49–1.23)	0.692	0.233
Model 2	1.0	1.35 (0.81–2.24)	1.06 (0.61–1.84)	0.87 (0.48–1.59)	0.436	0.540
**DPA (C 22:5 n – 3)**						
Model 1	1.0	0.78 (0.52–1.17)	0.58 (0.37–0.91)	0.64 (0.40–1.00)	0.076	0.019
Model 2	1.0	0.86 (0.52–1.43)	0.77 (0.45–1.30)	0.77 (0.44–1.34)	0.739	0.298
**DHA (C 22:6 n – 3)**						
Model 1	1.0	1.13 (0.75–1.69)	0.77 (0.50–1.20)	0.52 (0.32–0.86)	0.014	0.004
Model 2	1.0	1.29 (0.78–2.13)	0.99 (0.57–1.71)	0.63 (0.33–1.19)	0.124	0.120
**EPA + DHA**						
Model 1	1.0	0.91 (0.60–1.38)	0.73 (0.47–1.14)	0.62 (0.39–0.99)	0.182	0.028
Model 2	1.0	1.35 (0.82–2.22)	1.02 (0.58–1.80)	0.79 (0.43–1.45)	0.285	0.348
**EPA + DPA + DHA**						
Model 1	1.0	0.96 (0.64–1.45)	0.77 (0.50–1.19)	0.59 (0.36–0.95)	0.120	0.019
Model 2	1.0	1.56 (0.95–2.58)	1.17 (0.67–2.04)	0.76 (0.40–1.43)	0.07	0.303
**N – 3/n – 6 ratio**						
Model 1	1.0	0.88 (0.57–1.36)	0.96 (0.63–1.48)	0.72 (0.45–1.16)	0.559	0.258
Model 2	1.0	1.13 (0.68–1.86)	1.03 (0.61–1.74)	0.75 (0.42–1.33)	0.553	0.341

Model 1: adjusted for age, BMI, initial event, gender.

Model 2: adjusted for covariates in Model 1 + treatment allocation group (both placebos, B vitamin and placebo of omega 3, omega 3 and placebo of B vitamins, omega 3 and B vitamins), tobacco smoking, alcohol consumption, systolic blood pressure, diabetes status, plasma cholesterol (HDL and LDL), triglycerides, total fatty acid intake, n-3 supplementation and homocysteinemia.

**Table 4:** Multivariate hazard ratios (95% confidence intervals) showing the associations between baseline plasma lipid profile and incident cardiac events in the SU.FOL.OM3 study.

Plasma lipid profile	HR Q1	HR Q2	HR Q3	HR Q4	P	P for trend
**Total fatty acids**						
Model 1	1.0	1.12 (0.84–1.51)	1.23 (0.93–1.65)	1.23 (0.92–1.65)	0.452	0.130
Model 2	1.0	0.97 (0.67–1.42)	1.17 (0.73–1.87)	1.00 (0.47–2.13)	0.670	0.635
**Total SFA**						
Model 1	1.0	0.82 (0.62–1.10)	0.90 (0.68–1.19)	0.89 (0.67–1.18)	0.623	0.540
Model 2	1.0	0.76 (0.55–1.06)	0.90 (0.65–1.24)	0.77 (0.53–1.12)	0.339	0.301
**Palmitic acid (C 16:0)**						
Model 1	1.0	0.78 (0.59–1.05)	0.85 (0.64–1.13)	0.85 (0.64–1.13)	0.401	0.362
Model 2	1.0	0.70 (0.50–0.97)	0.81 (0.59–1.12)	0.76 (0.52–1.10)	0.183	0.209
**Trans fatty acids**						
Model 1	1.0	0.99 (0.75–1.31)	1.00 (0.75–1.34)	0.98 (0.73–1.31)	0.998	0.905
Model 2	1.0	0.97 (0.71–1.33)	1.02 (0.74–1.40)	0.94 (0.67–1.31)	0.966	0.789
**Total MUFA**						
Model 1	1.0	1.03 (0.77–1.37)	1.04 (0.78–1.39)	1.06 (0.79–1.41)	0.987	0.718
Model 2	1.0	1.02 (0.74–1.41)	1.05 (0.75–1.50)	1.13 (0.77–1.67)	0.932	0.535
**Oleic acid (C 18:1 n – 9)**						
Model 1	1.0	1.07 (0.80–1.42)	0.98 (0.73–1.31)	0.98 (0.73–1.31)	0.929	0.762
Model 2	1.0	1.06 (0.77–1.46)	0.97 (0.69–1.37)	1.00 (0.67–1.48)	0.959	0.873
**Total PUFA**						
Model 1	1.0	1.02 (0.77–1.35)	1.01 (0.76–1.34)	1.02 (0.76–1.36)	0.999	0.935
Model 2	1.0	1.10 (0.78–1.56)	1.08 (0.74–1.57)	1.10 (0.73–1.66)	0.957	0.710
**Total n-6 PUFA**						
Model 1	1.0	1.05 (0.79–1.39)	0.89 (0.66–1.20)	1.10 (0.83–1.46)	0.539	0.787
Model 2	1.0	1.10 (0.78–1.56)	0.93 (0.64–1.36)	1.16 (0.78–1.70)	0.594	0.650
**Linoleic acid (C 18:2 n – 6)**						
Model 1	1.0	0.75 (0.56–1.00)	0.84 (0.64–1.11)	0.84 (0.63–1.12)	0.261	0.357
Model 2	1.0	0.85 (0.62–1.18)	0.92 (0.68–1.26)	0.86 (0.62–1.20)	0.754	0.443
**Dihomo-Υ-linolenic acid (C 20:3 n – 6)**						
Model 1	1.0	0.88 (0.67–1.17)	0.74 (0.55–0.98)	0.93 (0.70–1.23)	0.209	0.342
Model 2	1.0	0.90 (0.68–1.19)	0.73 (0.54–0.98)	0.94 (0.71–1.29)	0.196	0.402
**Arachidonic acid (C 20:4 n – 6)**						
Model 1	1.0	0.87 (0.66–1.14)	0.80 (0.60–1.07)	0.86 (0.65–1.14)	0.467	0.227
Model 2	1.0	0.89 (0.64–1.23)	0.90 (0.64–1.26)	0.89 (0.62–1.28)	0.882	0.564
**Total n-3 PUFA**						
Model 1	1.0	0.99 (0.75–1.31)	0.82 (0.61–1.09)	0.89 (0.67–1.18)	0.480	0.241
Model 2	1.0	1.21 (0.88–1.67)	0.99 (0.71–1.40)	0.98 (0.70–1.38)	0.508	0.644
**α-linolenic acid (C 18:3 n – 3)**						
Model 1	1.0	0.87 (0.65–1.17)	0.96 (0.72–1.28)	1.15 (0.87–1.51)	0.287	0.253
Model 2	1.0	0.90 (0.64–1.26)	1.04 (0.75–1.46)	1.28 (0.93–1.77)	0.172	0.079
**Stearidonic acid (C 18:4 n – 3)**						
Model 1	1.0	1.10 (0.84–1.44)	0.83 (0.64–1.08)	0.67 (0.49–0.92)	0.013	0.005
Model 2	1.0	0.94 (0.69–1.29)	0.86 (0.65–1.15)	0.63 (0.44–0.90)	0.084	0.016
**EPA (C 20:5 n – 3)**						
Model 1	1.0	0.91 (0.70–1.20)	0.68 (0.51–0.91)	0.79 (0.59–1.04)	0.054	0.027
Model 2	1.0	1.05 (0.77–1.43)	0.82 (0.59–1.15)	0.80 (0.56–1.13)	0.292	0.102
**DPA (C 22:5 n – 3)**						
Model 1	1.0	0.88 (0.66–1.15)	0.82 (0.62–1.08)	0.79 (0.60–1.06)	0.365	0.091
Model 2	1.0	0.84 (0.61–1.15)	0.87 (0.63–1.19)	0.78 (0.56–1.09)	0.494	0.177
**DHA (C 22:6 n – 3)**						
Model 1	1.0	1.11 (0.84–1.47)	1.03 (0.77–1.37)	0.74 (0.55–1.00)	0.0480	0.049
Model 2	1.0	1.34 (0.97–1.86)	1.25 (0.87–1.76)	0.84 (0.58–1.21)	0.0206	0.249
**EPA + DHA**						
Model 1	1.0	0.90 (0.68–1.18)	0.81 (0.61–1.08)	0.82 (0.61–1.08)	0.426	0.122
Model 2	1.0	1.14 (0.82–1.57)	1.01 (0.72–1.42)	0.92 (0.65–1.30)	0.643	0.500
**EPA + DPA + DHA**						
Model 1	1.0	0.95 (0.72–1.26)	0.82 (0.61–1.09)	0.83 (0.62–1.10)	0.409	0.117
Model 2	1.0	1.21 (0.88–1.67)	1.02 (0.71–1.41)	0.93 (0.66–1.33)	0.412	0.464
**N – 3/n – 6 ratio**						
Model 1	1.0	0.71 (0.53–0.94)	0.73 (0.55–0.96)	0.80 (0.60–1.05)	0.0566	0.127
Model 2	1.0	0.81 (0.59–1.12)	0.85 (0.61–1.17)	0.85 (0.62–1.17)	0.582	0.377

Model 1: adjusted for age, BMI, initial event, gender.

Model 2: adjusted for covariates in Model 1 + treatment allocation group (both placebos, B vitamin and placebo of omega 3, omega 3 and placebo of B vitamins, omega 3 and B vitamins),, tobacco smoking, alcohol consumption, systolic blood pressure, diabetes status, plasma cholesterol (HDL and LDL), triglycerides, total fatty acid intake, n-3 supplementation and homocysteinemia.

**Table 5:** Multivariate hazard ratios (95% confidence intervals) showing the associations between baseline plasma lipid profile and incident cerebrovascular events in the SU.FOL.OM3 study.

Plasma lipid profile	HR Q1	HR Q2	HR Q3	HR Q4	P	P for trend
**Total fatty acids**						
Model 1	1.0	1.42 (0.73–2.79)	1.55 (0.80–3.00)	1.49 (0.77–2.91)	0.589	0.255
Model 2	1.0	0.47 (0.58–3.71)	1.61 (0.52–4.98)	1.27 (0.23–6.94)	0.691	0.621
**Total SFA**						
Model 1	1.0	0.70 (0.39–1.26)	0.72 (0.40–1.30)	0.69 (0.38–1.26)	0.539	0.252
Model 2	1.0	0.71 (0.36–1.41)	0.85 (0.43–1.69)	0.53 (0.22–1.29)	0.496	0.246
**Palmitic acid (C 16:0)**						
Model 1	1.0	0.86 (0.47–1.57)	0.85 (0.47–1.55)	0.83 (0.45–1.55)	0.932	0.578
Model 2	1.0	0.78 (0.38–1.61)	0.94 (0.46–1.90)	0.71 (0.29–1.72)	0.822	0.597
**Trans fatty acids**						
Model 1	1.0	1.05 (0.59–1.87)	0.79 (0.41–1.49)	0.90 (0.49–1.65)	0.806	0.539
Model 2	1.0	0.93 (0.48–1.83)	0.67 (0.31–1.46)	1.02 (0.50–2.08)	0.721	0.820
**Total MUFA**						
Model 1	1.0	1.84 (0.97–3.48)	1.24 (0.63–2.45)	1.93 (1.01–3.67)	0.127	0.141
Model 2	1.0	1.48 (0.71–3.05)	1.25 (0.58–2.67)	1.59 (0.67–3.82)	0.677	0.388
**Oleic acid (C 18:1 n – 9)**						
Model 1	1.0	0.81 (0.43–1.51)	0.99 (0.55–1.77)	1.09 (0.61–1.95)	0.931	0.672
Model 2	1.0	0.78 (0.38–1.58)	0.74 (0.36–1.52)	0.79 (0.34–1.83)	0.847	0.505
**Total PUFA**						
Model 1	1.0	0.81 (0.44–1.49)	1.02 (0.57–1.84)	0.85 (0.46–1.56)	0.840	0.782
Model 2	1.0	1.26 (0.56–2.85)	1.32 (0.55–3.18)	1.27 (0.50–3.27)	0.933	0.673
**Total n-6 PUFA**						
Model 1	1.0	1.05 (0.57–1.93)	1.12 (0.62–2.03)	0.86 (0.46–1.62)	0.855	0.703
Model 2	1.0	1.00 (0.45–2.23)	1.22 (0.54–2.75)	1.05 (0.44–2.51)	0.948	0.826
**Linoleic acid (C 18:2 n – 6)**						
Model 1	1.0	0.98 (0.54–1.77)	1.12 (0.62–2.01)	1.09 (0.59–2.01)	0.970	0.698
Model 2	1.0	0.93 (0.47–1.86)	0.64 (0.30–1.35)	1.03 (0.53–2.03)	0.624	0.789
**Dihomo-Υ-linolenic acid (C 20:3 n – 6)**						
Model 1	1.0	0.96 (0.51–1.82)	1.11 (0.60–2.05)	1.35 (0.75–2.45)	0.675	0.275
Model 2	1.0	1.15 (0.51–2.59)	1.44 (0.66–3.13)	1.87 (0.88–3.98)	0.357	0.077
**Arachidonic acid (C 20:4 n – 6)**						
Model 1	1.0	0.68 (0.39–1.16)	0.51 (0.28–0.92)	0.46 (0.24–0.86)	0.04	0.006
Model 2	1.0	0.69 (0.35–1.37)	0.52 (0.25–1.08)	0.44 (0.20–0.99)	0.18	0.033
**Total n-3 PUFA**						
Model 1	1.0	1.04 (0.60–1.79)	0.69 (0.37–1.27)	0.69 (0.36–1.29)	0.37	0.125
Model 2	1.0	1.80 (0.94–3.46)	0.80 (0.36–1.77)	0.79 (0.35–1.77)	0.05	0.245
**α-linolenic acid (C 18:3 n – 3)**						
Model 1	1.0	1.09 (0.62–1.93)	0.98 (0.54–1.77)	0.90 (0.49–1.68)	0.95	0.7
Model 2	1.0	0.95 (0.48–1.88)	0.87 (0.43–1.77)	0.94 (0.46–1.94)	0.98	0.80
**Stearidonic acid (C 18:4 n – 3)**						
Model 1	1.0	0.90 (0.54–1.49)	0.43 (0.23–0.80)	0.44 (0.22–0.89)	0.014	0.002
Model 2	1.0	0.76 (0.41–1.40)	0.38 (0.18–0.82)	0.50 (0.23–1.08)	0.058	0.014
**EPA (C 20:5 n – 3)**						
Model 1	1.0	1.08 (0.61–1.94)	1.14 (0.64–2.03)	0.83 (0.44–1.57)	0.78	0.68
Model 2	1.0	1.40 (0.69–2.82)	1.28 (0.63–2.61)	0.84 (0.37–1.91)	0.53	0.72
**DPA (C 22:5 n – 3)**						
Model 1	1.0	0.86 (0.49–1.51)	0.83 (0.47–1.47)	0.62 (0.33–1.17)	0.54	0.16
Model 2	1.0	0.91 (0.45–1.83)	1.05 (0.54–2.05)	0.59 (0.26–1.31)	0.50	0.31
**DHA (C 22:6 n – 3)**						
Model 1	1.0	1.06 (0.61–1.84)	0.65 (0.36–1.19)	0.64 (0.34–1.20)	0.22	0.070
Model 2	1.0	1.04 (0.52–2.09)	0.90 (0.44–1.86)	0.65 (0.29–1.47)	0.68	0.29
**EPA + DHA**						
Model 1	1.0	0.93 (0.52–1.64)	0.86 (0.48–1.53)	0.68 (0.36–1.27)	0.67	0.23
Model 2	1.0	1.21 (0.60–2.41)	1.12 (0.54–2.31)	0.73 (0.33–1.63)	0.62	0.47
**EPA + DPA + DHA**						
Model 1	1.0	0.98 (0.56–1.71)	0.79 (0.44–1.42)	0.63 (0.33–1.19)	0.47	0.12
Model 2	1.0	1.48 (0.75–2.91)	1.06 (0.51–2.23)	0.69 (0.30–1.60)	0.27	0.31
**N – 3/n – 6 ratio**						
Model 1	1.0	0.88 (0.48–1.59)	1.15 (0.66–2.00)	0.65 (0.34–1.26)	0.37	0.41
Model 2	1.0	1.13 (0.57–2.25)	1.22 (0.62–2.42)	0.71 (0.32–1.57)	0.56	0.54

Model 1: adjusted for age, BMI, initial event, gender.

Model 2: adjusted for covariates in Model 1 + treatment allocation group (both placebos, B vitamin and placebo of omega 3, omega 3 and placebo of B vitamins, omega 3 and B vitamins),, tobacco smoking, alcohol consumption, systolic blood pressure, diabetes status, plasma cholesterol (HDL and LDL), triglycerides, total fatty acid intake, n-3 supplementation and homocysteinemia.

In model 2, only stearidonic acid remained inversely associated with the risk of developing a major cardiovascular event, a cardiac event or a cerebrovascular event, while arachidonic acid was inversely associated with the risk of cerebrovascular event.

## Discussion

In this ancillary analysis which included patients with established and treated CVDs, plasma stearidonic acid and arachidonic acid showed protective effects on the recurrence of CVD and cerebrovascular diseases, respectively. The associations between total PFA, total n–3 PUFA, EPA, DPA, DHA, EPA + DHA, EPA + DPA + DHA and either major cardiovascular, cardiac or cerebrovascular events seemed to be mediated by established cardiovascular risks factors.

Numerous mechanistic studies have found that stearidonic acid favourably compares with dietary EPA in side-by-side experiments. Similar physiological effects were observed in ex vivo platelet aggregation studies, changes in tissue arachidonic acid content and eicosanoid formation, biomarkers of inflammation and modification of plasma lipid profiles. However, no epidemiological cohort study has tested the association between stearidonic acid with CVD. Moreover, stearidonic acid concentration is very low and at near the limit of detection, and dietary intakes is very low, thus the implications from a nutritional standpoint may be very limited. We also found evidence of an inverse association between arachidonic acid and CVD. Several studies [19]–[21] have shown an inverse association between plasma arachidonic acid and risk of CVD, while others have shown either no association [3], [22]. Higher Δ^5^-desaturase index (a ratio of arachidonic to dihomo γ-linolenic ratio) has been reported to be associated with higher insulin sensitivity [23], [24], and lower incidence of the metabolic syndrome [25] or CVD [26], which may partly explain the inverse association between arachidonic acid and cardiovascular events observed here. However, these results contrast with other mechanistic studies revealing the prothrombotic and proinflammatory properties of arachidonic acid, and merit further exploration [27]–[30].

Our results suggest that associations with long chain n–3 PUFA are mainly mediated by confounding factors. Contrary to our findings, most [1], [6], [22], [31], [32], but not all [33]–[35] of the observational cohort studies show an inverse association between fish consumption, fish oil supplementation or plasma values of fish derived long chain n–3 PUFA and CVDs. A meta-analysis of such cohort studies [1] concluded that there was a modest evidence of an inverse association between n–3 PUFA intake and CVD, with no evidence of a linear dose-response relationship. Despite adjustment for potential confounders, residual confounding is likely to have occurred in some of these observational cohort studies. Indeed, participants who consumed more PUFA were more likely to be non-smokers [33], [36]–[39], to be more physically active [33], [37], [38], to take dietary vitamins [33], to eat more dietary fibre, fruit and vegetables, and less saturated and trans fats [33], and to drink less alcohol [33], [37], [38]. Not only did all these studies not take into account such potential confounding factors, but also, adjusting for these covariables does not fully protect against residual confounding. Whether fish consumption provides other beneficial nutrients not present in pure fish oil remains questionable. The most recently published meta-analyses of randomized controlled trials showed conflicting results regarding the effects of fish oil or fish supplements on the occurrence of primary CVD. Leon et al [8] reported on the effects of fish oil (EPA and DPA) on mortality and arrhythmias from 12 studies including 32,779 patients. No beneficial effect on arrhythmic events or all-cause mortality was reported but a significant reduction in deaths from cardiac causes was found. Marik et al [9] identified 11 studies that included 39,044 patients. They found that dietary supplementation with n–3 PUFA significantly reduced the risk of cardiovascular deaths, sudden cardiac deaths, and non-fatal cardiovascular events. With a total of 29 randomized controlled trials (n = 35,144), with 25 of them concerning mortality and 14 restenosis, Filion et al [7] found that n–3 PUFA were not associated with mortality or with restenosis, even though the probability of some benefit remained high (0.93 and 0.90, respectively). Of the total population included in these meta-analyses, 20 to 35% were participants in the Gruppo Italiano per lo Studio della Sopravvivenza nell'Intarto Miocardio (GISSI)-Prevenzione trial [40]. In this trial, the treatment under investigation was not blinded, and only a low or moderate proportion of patients received modern intervention and medical treatment. Also, only 5.0% of the patients had coronary revascularisation at baseline, and only 4.7% were on cholesterol-lowering drugs at hospital discharge. Therefore, it is not clear whether the conclusions from these meta-analyses are still valid in 2011. Moreover, three doubled-blind randomized controlled trials [15], [18], [41], not included in the previous meta-analyses, where almost 20,000 patients participated for a median follow up over 4 years, failed to demonstrate a protective effect of n–3 PUFA supplementation on the reduction of major cardiovascular events in patients with personal history of CVD or prediabetes. The lack of association between baseline PFA and subsequent cardiovascular events in our study as well as in these two randomised controlled trials [15], [18], [41] could be due to an improvement in the efficacy of cardioprotective drug treatments between the 1995–1996 and 2006–2007 [42]. There been improvement in survival but also the causes of death have shifted from CV to non-cardiovascular origin [43]. Consequently, among patients who have had a CVD but who are receiving good clinical care and are at relatively low risk of future CV events, a beneficial effect of habitual dietary intakes of fatty acids may be difficult to detect [44] as illustrated by findings from the Omega trial [15].

The principal strength of this study is its large, well-characterized population with close to complete follow-up with respect to clinical endpoints. Also, we adjusted for most traditional risks factors, therapeutic parameters, and biomarkers characterizing CVD. We evaluated not only the clinical effects of widely studied long chains PUFA (EPA and DHA), but also the effects of a vast majority of plasma fatty acids. Despite adjustment for potential confounders, residual confounding is likely to have occurred in that observational analysis. A major potential confounder is engagement in physical activity, which was not available for our participants at baseline. As subjects with high levels of physical activity eat significantly more fish compared with their more sedentary counterparts [33], [37], [38], it is likely that adjusting for levels of physical activity might have reduced the association between arachidonic acid or stearidonic acid and cardiovascular outcomes.

In our population with established and well-treated history of ischemic heart disease or stroke, the inverse association of long chain n–3 PUFA with CVD was mainly driven by well-known cardiovascular risk factors. These results, as the lack of impact of our supplementation trial with DHA-EPA on CVD recurrence [18], do not support the recommendations of use of n-3 PUFA for the secondary prevention of CVD.

## Supporting Information

Checklist S1 CONSORT Checklist.
(DOC)
